# The Effects of Warm Acupuncture on the Expression of *AMPK* in High-Fat Diet-Induced MAFLD Rats

**DOI:** 10.3390/cimb46100687

**Published:** 2024-10-17

**Authors:** Yumi Lee, Donghee Choi, Junghye Park, Jae Gwan Kim, Taejin Choi, Daehwan Youn

**Affiliations:** 1 Department of Biomedical Science and Engineering, Gwangju Institute of Science and Technology, Gwangju 61005, Republic of Korea; leeyumi01@gist.ac.kr (Y.L.); wnsla1052@naver.com (J.P.); jaekim@gist.ac.kr (J.G.K.); 2 Department of Korean Medicine, Dongshin University, Naju 58245, Republic of Korea; 1004cdh@hanmail.net; 3 DongHaeng Convalescent Hospital, Gwangju 61251, Republic of Korea; public21@hanmail.net

**Keywords:** MAFLD, lipid synthesis, warm acupuncture, *AMPK*, *SREBP1*, *ACC*

## Abstract

This study investigated the effects of acupuncture and warm acupuncture on the expression and mechanism of the AMP-activated protein kinase (*AMPK*) signalling pathway associated with lipid accumulation in the liver tissue of rats with metabolic dysfunction-associated fatty liver disease (MAFLD) induced by a high-fat diet. Sprague–Dawley rats were categorised into four groups: control (CON), untreated MAFLD (MAFLD), and two MAFLD groups treated with acupuncture (ACU) and warm acupuncture (WA). The treatment groups underwent 16 application sessions over 8 weeks at the SP9 and BL18 acupoints. We measured the expression levels of *AMPK*, sterol regulatory element-binding protein1 (*SREBP1*), acetyl-coenzyme A carboxylase (*ACC*), peroxisome proliferator-activated receptorα (*PPARα*), carnitine palmitoyltransferase1 (*CPT1*), and *CPT2*. *AMPK* was activated in both ACU and WA groups. WA downregulated both SREBP1 and ACC expression at the protein level, whereas the acupuncture treatment downregulated SREBP1 expression. Additionally, WA selectively induced the activation of signalling pathways related to *AMPK, PPARα, CPT1*, and *CPT2* at the mRNA level. Histological observations confirmed that fat accumulation was reduced in both the ACU and the WA groups compared to the MAFLD group. The WA treatment-promoted amelioration of HFD-induced MAFLD may be related to the activation of the *AMPK/SREBP1/ACC* pathway in the liver.

## 1. Introduction

Metabolic dysfunction-associated fatty liver disease (MAFLD), previously known as non-alcoholic fatty liver disease (NAFLD), affects around a quarter of the adult population globally, presenting a substantial health and economic burden to societies and lacking any approved therapy [1]. It spans a spectrum of conditions, from simple steatosis to non-alcoholic steatohepatitis (NASH), involving inflammation and liver cell damage, potentially progressing to varying degrees of cirrhosis and liver failure. Recent studies on MAFLD aim to enhance our understanding of the condition, improve diagnostics, and develop novel treatments. These studies have focused on mitochondrial dysfunction [2], insulin resistance and lipid metabolism [3], inflammation [4], and genetic and epigenetic regulation [5]. Moreover, most current studies have focused on deciphering the mechanisms linking mitochondria to MAFLD, offering valuable insights into the multifactorial nature of MAFLD pathophysiology and identifying potential therapeutic targets for the prevention and treatment of this increasingly prevalent liver disease [2,6]. The lipid synthesis mechanism in MAFLD is complex and involves a network of hormonal signals, transcription factors, and enzymatic pathways [7,8].

AMP-activated protein kinase (*AMPK*) is an energy sensor and metabolic regulator that responds to the hormonal and nutritional status of the body. This enzyme has recently gained significant attention owing to its ability to regulate various metabolic pathways, including hepatic lipid metabolism. *AMPK* plays a crucial role in regulating hepatic lipid metabolism by mediating the synthesis and breakdown of fatty acids [9]. The activation of *AMPK* leads to the phosphorylation and inactivation of acetyl-CoA carboxylase (*ACC*), an enzyme essential for ATP-consuming processes, such as fatty acid and cholesterol synthesis. In a previous study, liver-specific *AMPK* deletion reportedly increased the blood triglyceride levels and enhanced lipid synthesis in the liver [10].

The *AMPK* signalling pathway is closely related to the functions of sterol regulatory element-binding protein 1 (*SREBP1*) and proliferator-activated receptor α (*PPARα*), which regulate fatty acid synthesis and oxidation. Both *SREBP1* and *PPARα* are key downstream effectors of lipid metabolism involving *AMPK*, making *AMPK* a significant therapeutic target for the treatment of obesity and MAFLD. In previous studies, the overexpression of *SREBP1* and its downstream gene *ACC*, which downregulates *AMPK,* was shown to induce increased de novo lipogenesis (DNL) and cause fatty liver disease in mice. Additionally, this overexpression downregulates the expression of *PPARα* and its downstream genes carnitine palmitoyltransferase 1 (*CPT1*) and *CPT2*, highlighting another mechanism involved in the development of fatty liver disease [11].

Multiple clinical trials and meta-analyses have investigated acupuncture as a potential complementary therapy for MAFLD, suggesting that it may enhance liver function by improving the blood flow and promoting liver regeneration. Reports indicate that acupuncture can improve the levels of liver enzymes, such as alanine transaminase (ALT) and aspartate transaminase (AST) and address lipid metabolism abnormalities, improve liver function, reduce simple steatosis, decrease abdominal fat, and improve other anthropometric parameters. These improvements have been observed after treatments such as electroacupuncture (EA), manual acupuncture, and acupoint injections in patients with MAFLD [12].

Several experimental studies have shown that the *AMPK/ACC* pathway is a key target of EA in regulating metabolic diseases. Zang et al. [13] suggested that repeated EA therapy could improve diet-induced insulin resistance, possibly by activating *AMPK* signalling in skeletal muscles. In their study, the phospho-AMPKα levels were significantly higher in animals receiving EA than in control animals immediately after stimulation. Similarly, Li et al. [14] demonstrated that EA positively regulated the *AMPK/ACC* pathway by reducing ACC gene expression and increasing AMPK gene expression in high-fat diet (HFD)-induced insulin-resistant rats, particularly in muscle tissues.

However, the effects of acupuncture on *AMPK* and associated proteins in the liver remain unclear. Despite its evident clinical benefits, the molecular mechanisms by which acupuncture promotes recovery in patients with MAFLD remain to be fully elucidated.

In this context, the present study investigated the effects of acupuncture and warm acupuncture (WA) treatments on the *SREBP1* and *PPARα* pathways—key signalling pathways related to *AMPK* in the liver—using an HFD-induced MAFLD rat model. Additionally, we focused on morphological and functional changes in the liver.

## 2. Materials and Methods

### 2.1. Animals

Pathogen-free male Sprague–Dawley (SD) rats, weighing between 140 and 160 g, were housed under controlled conditions of temperature (23 ± 1 °C) and humidity (60 ± 5%), with access to food (Samtaco, Osan, Republic of Korea) and tap water. All animal care and experimental protocols were approved by the Animal Management and Use Commission of Dongshin University (approval numbers: DSU-2022-01-03 and DSU-2023-01-02).

### 2.2. Induction of MAFLD and Grouping

Following a 7-day acclimatisation period, 20 male SD rats were randomly divided into a non-treated control group (CON, *n* = 5) and an experimental group (*n* = 15) for MAFLD induction. The control group maintained a general diet (Samtaco, Osan, Republic of Korea), and the experimental group was fed a 45% HFD (Research Diet, NJ, USA) supplemented with 10% fructose (Samchun, Pyeongtaek, Republic of Korea) in water for 20 weeks. At the 12-week mark, the triglyceride (TG) levels were measured to confirm MAFLD, after which the rats were further divided into three groups: a MAFLD group (MAFLD, *n* = 5), an acupuncture treatment group (ACU, *n* = 5), and a WA treatment group (WA, *n* = 5).

### 2.3. Acupuncture and WA Stimulation

Acupuncture was administered at the SP9 and BL18 acupoints for 5 min each, whereas the WA treatment involved applying moxibustion to needle springs at the same acupoints for 5 min each. The acupoint locations were determined according to international standards [15]. The ACU and WA groups received their respective treatments twice a week for 8 weeks, for a total of 16 sessions. Both the control and the MAFLD groups were subjected to food and water intake restrictions for the same duration as the acupuncture and warm acupuncture groups, thereby eliminating any group-specific bias.

### 2.4. Sample Collection

Upon completion of the treatment, blood was collected through cardiac puncture under respiratory anaesthesia, the serum was separated, and the liver was isolated. Liver tissues for tissue observation were stored in 10% formalin solution, and liver tissues for qRT-PCR and Western blotting analysis were stored at −80 °C.

### 2.5. Body and Liver Weight

The body weight gain for each rat was measured using a balance (Cas, Yangju, Republic of Korea) and calculated as the difference in body weight from the beginning of the diet to the end of the 20-week period. At the end of the experiment, the animals were sacrificed, and their liver weight was measured.

### 2.6. Biochemical Analysis of Serum Enzyme Levels

The serum ALT, AST, and TG levels were measured using a Fuji Dri-Chem Clinical Chemistry Analyser (Fujifilm, Tokyo, Japan).

### 2.7. RNA Isolation and qRT-PCR

Total RNA was extracted from the liver tissues (50 mg) using the TRIzol isolation reagent (Thermo Fisher Scientific, Waltham, MA, USA). The extracted RNA was quantified using a NanoDrop spectrophotometer (Thermo Fisher Scientific, Waltham, MA, USA). Following quantification, the RNA was reverse-transcribed into cDNA using a cDNA synthesis master mix (LeGene Biosciences, San Diego, CA, USA). Real-time PCR was performed on a CFX Connect Real-Time PCR Detection System (Bio-Rad, Hercules, CA, USA) using the SB-Green qPCR Master Mix (LeGene Biosciences, San Diego, CA, USA). The sequences of the following genes were analysed: *AMPK*, *SREBP1*, *ACC*, *PPARα*, *CPT1*, and *CPT2*. The sequences of these genes are listed in Table 1. The results are expressed as fold change and were calculated using the comparative 2^−ΔΔCT^ method.

### 2.8. Western Blotting

The liver tissues (50 mg) were lysed using a protein extraction solution (IntronBio, Sungnam, Republic of Korea). Proteins were quantified using a bicinchoninic acid assay kit (Thermo Fisher Scientific, Waltham, MA, USA). Following quantification, the proteins were loaded and separated by sodium dodecyl sulphate–polyacrylamide gel electrophoresis. The proteins were then transferred onto polyvinylidene difluoride (PVDF) membranes. After blocking with 5% skim milk–TBST for 1 h at room temperature, the PVDF membranes were incubated overnight at 4 °C with primary antibodies. These included AMPK (1:500, Cell Signaling Technology, Danvers, MA, USA), SREBP1 (1:300, Bioss, Woburn, MA, USA), ACC (1:250, Cell Signaling Technology, Danvers, MA, USA), PPARα (1:500, Thermo Fisher Scientific, Waltham, MA, USA), CPT1 (1:300, Bioss, Woburn, MA, USA), CPT2 (1:800, Thermo Fisher Scientific, Waltham, MA, USA), and β-actin (1:1000, Thermo Fisher Scientific, Waltham, MA, USA) antibodies. The following day, the membranes were incubated for 1 h at 25 °C with a peroxidase-conjugated Affinipure goat anti-rabbit IgG antibody (1:1000, Jackson Immuno Research, West Grove, PA, USA). The intensity of the bands was quantified using an ImageQuant LAS 500 (GE Healthcare, Tokyo, Japan).

### 2.9. Histological Analysis

Liver tissue was fixed in 10% buffered formaldehyde and sectioned into 6 μm slices. Haematoxylin and eosin (H&E) (Sigma, St. Louis, MI, USA) and Masson’s trichrome staining (ScyTek Laboratories, West Logan, WV, USA) were performed. Oil Red O staining (Statlab, McKinney, TX, USA) was conducted on tissue samples embedded in frozen section compound (Leica, Richmond, VA, USA) and cut into 10 μm sections. Perilipin immunohistochemistry was performed by incubating the samples with a perilipin antibody (1:300, Thermo Fisher Scientific, Waltham, MA, USA), followed by counterstaining with haematoxylin. All the stained tissues were examined under a light microscope (Nikon, Tokyo, Japan).

### 2.10. Statistical Analysis

The data were analysed as the mean ± standard deviation (SD), and a nonparametric one-way ANOVA with Dunn’s multiple comparisons post-hoc test was used to assess statistical significance. All statistical analyses were performed using GraphPad Prism (version 8.4.1, GraphPad Software, Boston, MA, USA). Data from the experimental groups were compared with data from the control group at two significance levels: α = 0.05 (*p* < 0.05) and α = 0.01 (*p* < 0.01).

## 3. Results

### 3.1. Comparison of Body and Liver Weight and Biochemical Parameters

**Figure 1:**
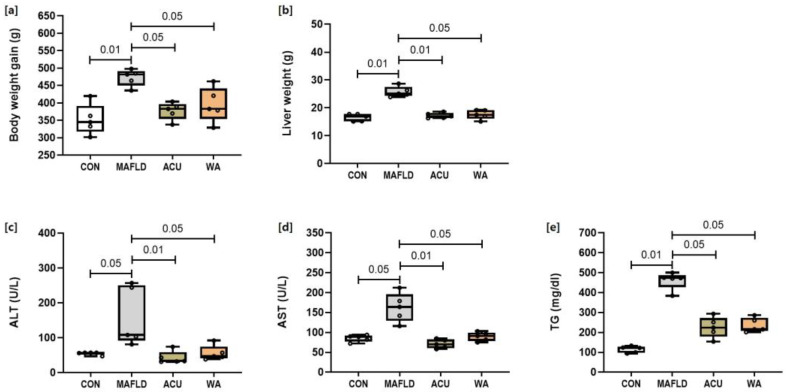
Changes in growth and serum biochemical parameters in response to acupuncture and warm acupuncture treatments in a MAFLD model were evaluated. Growth parameters include terminal body weight gain (**a**) and liver weight (**b**). Serum levels assessed are (**c**) alanine transaminase (ALT), (**d**) aspartate transaminase (AST), and (**e**) triglycerides (TG) levels. Data are presented as mean ± standard deviation (SD) for each group, with individual data points (dots) representing values from individual rats.

At the end of the 20-week experiment, body weight gain was calculated by subtracting the initial body weight from the final weight measured at the end of the period. The MAFLD group (*p* < 0.01) exhibited a significant increase in terminal body weight gain and liver weight compared to the CON group, whereas the ACU and WA groups showed a significant (*p* < 0.05, *p* < 0.01) decrease in body weight gain and liver weight compared to the MAFLD group (Figure 1a,b).

The MAFLD group showed a significant increase in serum ALT (*p* < 0.05), AST (*p* < 0.05) and TG (*p* < 0.01) levels compared with the CON group. The ACU and WA treatments in rats with MAFLD significantly (*p* < 0.05, *p* < 0.01) normalised serum ALT and AST activities and reduced the TG levels in the ACU and WA groups, although these values did not fully return to normal (Figure 1c–e).

### 3.2. Expression of mRNA Related to the AMPK Signalling Pathway

**Figure 2:**
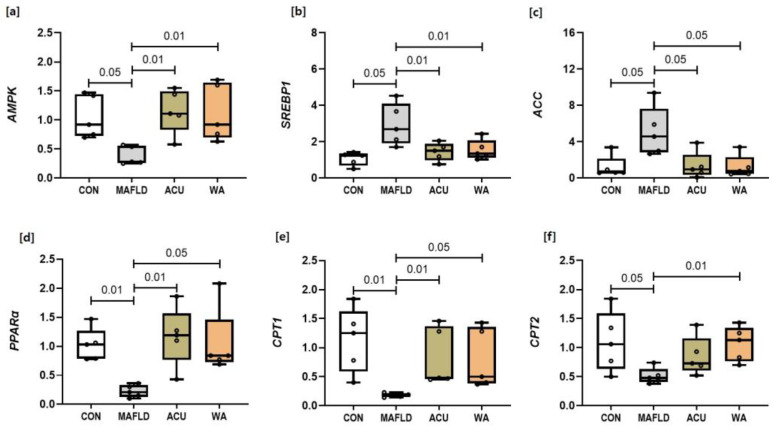
Changes in the mRNA expression levels of genes associated with the *AMPK* and *SREBP1/ACC* signalling pathways in the MAFLD model following acupuncture treatment. The expression levels of (**a**) AMP-activated protein kinase (*AMPK*), (**b**) sterol regulatory element-binding protein 1 (*SREBP1*), (**c**) acetyl-CoA carboxylase (*ACC*), (**d**) peroxisome proliferator activated receptor α (*PPARα*), (**e**) carnitine palmitoyltransferase 1 (*CPT1*), (**f**) carnitine palmitoyltransferase 2 (*CPT2*) mRNA. The measurements were conducted using qRT-PCR. The data are presented as mean ± standard deviation (SD) for each group, with individual data points (dots) representing values from individual rats.

The pathogenesis and therapeutic mechanisms of *AMPK*-associated MAFLD involve lipid synthesis and fatty acid oxidation. We analysed the genes associated with these processes to assess the effects of the acupuncture and WA treatments. The RT-qPCR results revealed that in the MAFLD group, *SREBP1* and *ACC* mRNA expression significantly (*p* < 0.05) increased compared to the CON group, while *AMPK* (*p* < 0.05), *PPARα* (*p* < 0.01), *CPT1* (*p* < 0.01), and *CPT2* (*p* < 0.05) mRNA expression significantly decreased, indicating impairment of the *AMPK* signalling pathway in MAFLD. Following the ACU and WA treatments, *AMPK, PPARα*, and *CPT1* mRNA expression in the liver tissues of MAFLD rats significantly (*p* < 0.05, *p* < 0.01) increased, leading to a significant decrease in *SREBP1* (*p* < 0.01) and *ACC* (*p* < 0.05) mRNA expression in both the ACU and the WA groups. However, *CPT2* mRNA expression increased significantly (*p* < 0.01) in the WA group. These findings suggest that acupuncture and WA exerted distinct therapeutic effects on MAFLD (Figure 2).

### 3.3. Expression of Proteins Related to the AMPK Signalling Pathway

**Figure 3:**
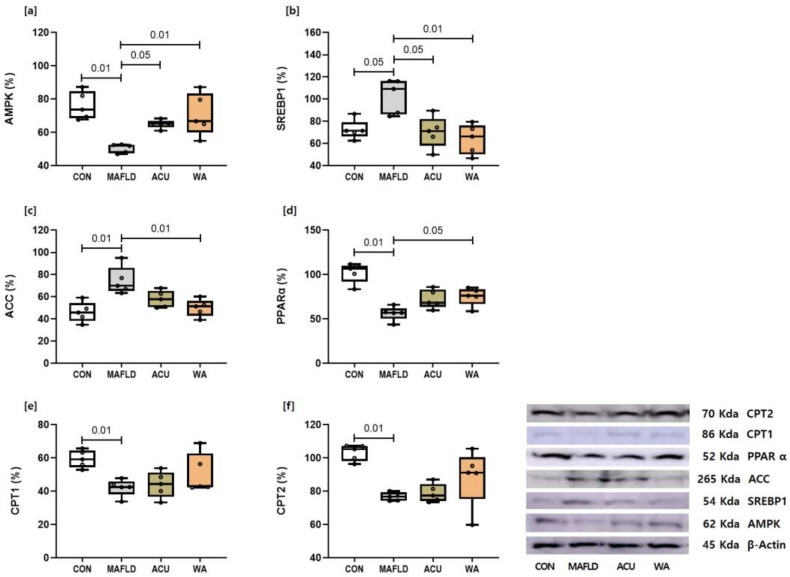
Changes in the expression of protein regulators in liver tissue related to the AMPK and SREBP1/ACC pathways in MAFLD models subjected to acupuncture. The proteins analysed by Western blot include (**a**) AMPK, (**b**) SREBP1, (**c**) ACC, (**d**) PPARα, (**e**) CPT1, and (**f**) CPT2. The data are presented as mean ± standard deviation (SD) for each group, with individual data points (dots) representing values from individual rats.

To further elucidate the mechanisms by which the AMPK-related acupuncture and WA treatments suppressed excessive fat accumulation in the liver, we analysed the key proteins involved in lipid metabolism in the liver tissue using Western blotting. As shown in Figure 3, the protein levels of AMPK, PPARα, CPT1, and CPT2 were significantly (*p* < 0.01) reduced, while those of SREBP1 (*p* < 0.05) and ACC (*p* < 0.01) were significantly increased in the MAFLD group compared to the CON group. Regarding the DNL process, which converts carbohydrates into fatty acids via the AMPK pathway, the protein expression levels of SREBP1 and ACC were significantly (*p* < 0.01) lower in the WA group than in the MAFLD group, whereas only SREBP1 protein expression was significantly (*p* < 0.05) lower in the ACU group. These findings suggest that the AMPK/SREBP1/ACC signalling pathway was restored by the WA treatment in MAFLD, whereas the AMPK/SREBP1 signalling pathway was restored by the acupuncture treatment (Figure 3). However, for AMPK pathway factors involved in mitochondrial β-oxidation, which primarily oxidises fatty acids, no significant changes were observed, contrasting with the mRNA results mentioned in Figure 2.

### 3.4. Histopathologic and Immunohistological Evaluations

**Figure 4:**
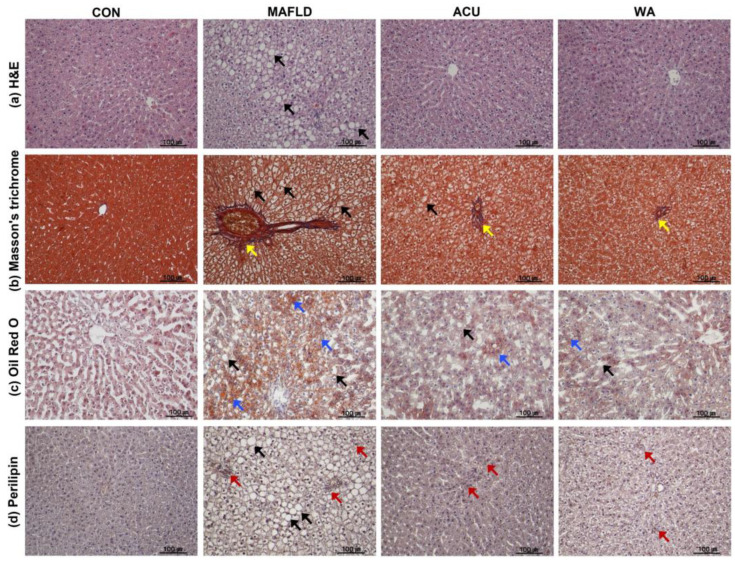
Histological changes in MAFLD models due to acupuncture treatments. The images include (**a**) haematoxylin and eosin (H&E) staining, (**b**) Masson’s trichrome staining for collagen fibres, (**c**) Oil Red O staining for lipid accumulation, and (**d**) perilipin immunohistochemistry for lipid droplet-associated protein, all captured at 200× magnification. Black arrow: lipid droplets; yellow arrow: fibrosis; blue arrow: lipid accumulation, red arrow: localized expression of perilipin.

H&E staining revealed macrovesicular steatosis and inflammatory cell infiltration in the tissues. The MAFLD group displayed increased cell expansion, inflammatory infiltration, and disarray of the liver lobule structures. However, both ACU and WA groups showed a significant reduction in inflammatory lesion infiltration, which improved the disordered liver lobule structure (Figure 4a). Masson’s trichrome staining indicated that the MAFLD group had larger and denser lipid droplets than the CON group. Fibrosis, marked by inflammatory infiltration, progressed in the MAFLD group. Conversely, both the ACU and the WA groups demonstrated an improvement in liver tissue fibrosis progression and a reduction in lipid droplet density compared with the MAFLD group (Figure 4b). Notably, the ACU group exhibited a significant decrease in lipid accumulation, as observed by Oil Red O staining, compared with the MAFLD group, which had denser lipid droplets (Figure 4c). Additionally, through the expression of perilipin, a key adipogenesis marker, we confirmed that the MAFLD group exhibited more severe steatosis than the CON group. In the ACU group, the perilipin expression level was notably reduced, suggesting that acupuncture regulated the production of lipid droplets in hepatocytes (Figure 4d).

## 4. Discussion

The liver is a major regulator of lipid homeostasis, regulating fatty acid absorption, synthesis, oxidative degradation, and lipid export and redistribution. Abnormalities in enzymes, gene regulatory elements, and molecular factors that maintain hepatic lipid homeostasis lead to abnormal lipid accumulation, which promotes the development of MAFLD [16].

Lipodystrophy, caused primarily by the inability of the adipose tissue to store fatty acids, can result in hepatic steatosis. Consequently, hepatocyte swelling due to lipid droplet accumulation is a hallmark of MAFLD [17]. The incidence of MAFLD, which is the most common liver disease in modern clinical practice, has increased due to the increasing obesity rates in developed countries influenced by a westernised culture. It affects approximately 30% of the adults in these countries and poses a significant healthcare concern. HFD-induced MAFLD models have been recognised as the most suitable animal models for understanding human MAFLD, forming the foundation of MAFLD research [18]. Excessive lipid accumulation in the liver tissue results from an imbalance between circulating lipid uptake, de novo lipogenesis, free fatty acid oxidation, and triglyceride-rich lipoprotein secretion. This imbalance triggers a cycle leading to lipid peroxidation, stress, and subsequent liver damage. Nuclear transcription factors, membrane transport proteins, and enzyme metabolism play diverse roles in different stages of MAFLD progression [17].

Figure 1 shows that the HFD-induced MAFLD group exhibited increased AST, ALT, and TG levels compared to the CON group. These levels also decreased in the treatment groups, confirming the successful establishment of the MAFLD model and indicating promising treatment outcomes. These findings align with those of previous studies using acupuncture in HFD-induced MAFLD rats, highlighting the impact of the acupuncture and WA treatments on the serum levels of these molecules [19,20].

*AMPK*, a cellular energy-sensing enzyme and a key regulatory factor in hepatic lipid homeostasis, plays a crucial role in MAFLD owing to its involvement in various aspects of lipid metabolism, such as the synthesis and oxidative degradation of fatty acids and triglycerides. As *AMPK* activity is reduced by obesity and diabetes, increasing its activity is considered a viable therapeutic strategy for treating MAFLD [19,21].

Recent studies have shown that *AMPK* activity is significantly affected by changes in the intracellular AMP/ATP ratio. When the AMP/ATP level increases, *AMPK* is activated, leading to the inhibition of lipogenesis and the promotion of fatty acid oxidation. As an important metabolic regulatory component, *AMPK* reflects the cellular stress levels under conditions of oxidative stress and energy deficiency and influences the lipid metabolism by phosphorylating target proteins. Previous studies demonstrated that once activated, *AMPK* phosphorylates a series of metabolic proteins involved in fatty acid synthesis, cholesterol synthesis, and fatty acid oxidation, thereby regulating the cellular lipid metabolism [22,23,24].

*AMPK* activation is a current treatment option for MAFLD. *AMPK* plays a key role in mediating the beneficial effects of phosphorylating downstream target proteins, reducing lipid accumulation, promoting fatty acid oxidation, and inhibiting cholesterol and fatty acid synthesis. Specifically, *AMPK* activation may reduce MAFLD by the following mechanisms: (1) suppressing DNL in the liver; (2) increasing fatty acid oxidation in the liver [21,22,23]. The objective of our study was to determine the effects of acupuncture and WA on the two primary mechanisms of *AMPK* activation in a high-fat diet-induced MAFLD rat model.

The first pathway involving *AMPK* is the *AMPK/SREBP1/ACC* axis, which is a part of the DNL process that converts carbohydrates into fatty acids. *AMPK* activation suppresses DNL by preventing the dimerisation of cytosolic ACC following *AMPK* phosphorylation, thereby decreasing ACC activity and regulating lipogenesis by reducing malonyl-CoA production [25].

The SREBP family, which includes *SREBP1a*, *SREBP1c*, and *SREBP2*, was originally identified as a group of transcription factors that regulates the expression of genes involved in fatty acid, TG, and cholesterol metabolism. SREBP1 is a transcription factor primarily expressed in the liver tissue, which controls the biosynthesis of cholesterol, fatty acids, and triglycerides. When *AMPK* is inactivated, *SREBP1* expression is upregulated, leading to the activation of mRNA and proteins, such as fatty acid synthase (*FAS*), acetyl-CoA carboxylase, acetyl-CoA synthase, and HMG-CoA reductase. This activation contributes to the regulation of the downstream enzyme *ACC*, which is involved in fatty acid synthesis and ultimately causes hepatic lipid metabolism disorders. This is now recognised as a major regulatory mechanism for changes in the intracellular lipid content [26,27,28,29,30].

Chiakang et al. [31] reported that *AMPK* promotes fatty acid oxidation and autophagy, inhibits cholesterol and fatty acid production, and reduces DNL via the *AMPK/SREBP1*-mediated pathway. Additionally, a previous study demonstrated that L-theanine, which regulates lipid metabolism by activating the *AMPK* signalling pathway in fatty acid-induced SD rats, inhibits lipid synthesis by activating AMPK through the *AMPK/SREBP1/ACC* pathway, significantly downregulating *ACC* phosphorylation and the expression level of *SREBP1* [32]. This suggests that, among the various regulatory mechanisms by which *AMPK* inhibits lipid and cholesterol synthesis, the *AMPK/SREBP1/ACC* pathway is particularly important. Therefore, the *AMPK* pathway helps to maintain the lipid metabolism in a stable state and protects against pathological conditions that can lead to fatty liver disease.

In a recent study on the effects of acupuncture on DNL, Yu et al. [33] reported that an EA intervention improved lipid metabolism disorders by reducing *SREBP1c* mRNA expression and the free fatty acid levels in MAFLD rats. Hong et al. [34] found that a WA treatment for >3 months alleviated MAFLD symptoms in patients. Building on this research, we evaluated the effect of thermal stimulation with acupuncture needles, rather than EA, on DNL-induced liver lipid accumulation due to DNL in an HFD-induced MAFLD model. *AMPK* mRNA expression increased in the liver tissue of MAFLD rats, whereas *SREBP1* and *ACC* mRNA expression decreased in both the ACU and the WA groups. Similarly, the protein expression levels of *SREBP1* and *ACC* were significantly lower in the WA group than in the MAFLD group, whereas only *SREBP1* protein expression was reduced in the ACU group. These findings suggest that WA treatments may effectively prevent HFD-induced lipid accumulation in the liver by activating the *AMPK* pathway. Additionally, the inhibition of the *SREBP1/ACC* signalling pathway by the WA treatment appears to be closely associated with *AMPK* activation.

The second pathway related to *AMPK* is the *AMPK/PPARα/CPT1,2* axis, a mitochondrial β-oxidation pathway that primarily oxidises short-, medium-, and long-chain fatty acids. When fatty acids enter a healthy liver tissue, they are transported from the cytoplasm to the mitochondrial matrix by *CPT1* and *CPT2*, where they are broken down into acetyl-CoA and fully oxidised through a series of biochemical reactions, including the tricarboxylic acid cycle. During the regulation of this mitochondrial β-oxidation, *AMPK* plays a crucial role in maintaining the balance of lipid metabolism. However, when this molecular pathway is disrupted, medium-chain and long-chain fatty acids cannot efficiently pass through the membrane to enter the mitochondria, directly relating *CPT1* and *CPT2* in the mitochondrial membrane to the efficiency of fatty acid oxidation [35,36].

Previous studies have shown that *AMPK* regulates *PPARα* activity and controls mitochondrial biogenesis and energy metabolism. *PPARα*, a key regulator of lipid metabolism and mitochondrial function in the liver, can have beneficial effects on fatty liver disease. The activation of *PPARα* promotes fatty acid oxidation and mitochondrial biogenesis and reduces inflammation. Furthermore, it encourages the transcription of genes involved in fatty acid oxidation, including enzymes such as *CPT1*, which facilitates fatty acid transport into the mitochondria for oxidation [37]. By enhancing fatty acid oxidation, *PPARα* activation aids in reducing TG and lipid droplet accumulation in hepatocytes, thereby mitigating fatty liver disease.

In this regard, García-Villafranca et al. [38]. investigated whether *AMPK* plays a role in the development of ethanol-induced fatty liver disease. Their results showed that chronic ethanol exposure decreased the expression and activity of *AMPK* and *CPT1* in hepatocytes, which were effectively reversed by treatment with the *AMPK* agonist *AICAR*. In another study, overexpression of *AMPKα1* in the human liver cell line L02 increased *CPT1* gene expression along with a decrease in lipid-loaded hepatocytes [39].

In this study, we examined the effects of acupuncture and WA treatments on the DNL pathway associated with *AMPK* and evaluated the metabolic factors related to mitochondrial lipid metabolism following *AMPK* activation. As shown in Figure 2, *CPT1* and *CPT2* mRNA expression increased in the WA group, while *CPT2* mRNA expression increased only in the ACU group via *PPARα* activation. However, no significant changes in the CPT1 or CPT2 protein levels were observed in any of the experimental groups, as depicted in Figure 3. These findings suggest that while the WA treatment may not alter the levels of the proteins involved in lipid-lowering pathways, it may influence the mRNA expression of *PPARα*, *CPT1*, and *CPT2* within the mitochondria.

Histological examination is a vital diagnostic tool for liver-related diseases, particularly in clinical practice where hepatic steatosis is typically identified through imaging or histopathology [40]. In this study, we used H&E and Masson’s staining to observe pathological changes in the liver tissue. Oil Red O staining was used to evaluate lipid accumulation in hepatocytes. The results revealed a progressive worsening of liver pathology as MAFLD progressed. However, the acupuncture and WA treatments effectively ameliorated MAFLD, a finding that aligns with previous studies on this disease [41,42,43]. We also observed that perilipin, a protein known to be overexpressed in MAFLD and closely associated with obesity, was present on the surface of lipid droplets. Importantly, perilipin is closely associated with *SREBP* signalling [44]. Our evaluation of perilipin expression in the liver tissue revealed severe steatosis in the MAFLD group, whereas the treatment groups showed reduced expression levels, consistent with our mRNA and protein analysis results (Figure 4d).

**Figure 5:**
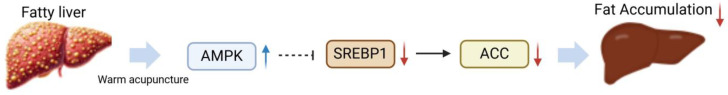
Mechanism of AMPK Activation via warm acupuncture in the alleviation of MAFLD.

By examining the representative pathways through which *AMPK* influenced fatty acid production following the acupuncture and WA treatments, we found that acupuncture primarily affected SREBP1, whereas the WA treatment negatively regulated both SREBP1 and ACC, thus suppressing fatty liver disease. Additionally, our study revealed that the WA treatment led to limited activation of signalling pathways involving *AMPK/PPARα/CPT1(2)* at the mRNA level, which differ from those involving SREBP1 and ACC. This suggests that further investigation of the mitochondria-related mechanisms is necessary. Therefore, WA treatments may serve as potential therapeutic approaches for lipid metabolism dysfunctions owing to their ability to activate *AMPK* (Figure 5).

## 5. Conclusions

This study suggests that WA treatments at the SP9 and BL18 acupoints can activate *AMPK*, downregulate the *SREBP1/ACC* pathway to ameliorate hepatic lipid accumulation, and improve HFD-induced liver function disturbances, highlighting their novel therapeutic potential for treating MAFLD.

## Figures and Tables

**Table 1:** The nucleotide primer sequences.

Target Gene	Primer Sequence
*GAPDH*	F: 5′-GGC ACA GTC AAG GCT GAG AAT G-3′ R: 5′-ATG GTG GTG AAG ACG CCA GTA-3′
*AMPK*	F: 5′-GCT CGC AGT GGC TTA TCA T-3′ R: 5′-TGG ACA GCG TGC TTT GG-3′
*SREBP1*	F: 5′-GGA CGA GCT ACC CTT CGG T-3′ R: 5′-CTG TCT CAC CCC CAG CAT AG-3′
*ACC*	F: 5′-CAC ATC ATG AAG GAG GAG G-3′ R: 5′-GCT ATC ACA CAG CCT GGG TC-3′
*PPARα*	F: 5′-TGC GGACTA CCA GTA CTT AGG G-3′ R: 5′-GCT GGA GAG AGG GTG TCT GT-3′
*CPT1*	F: 5′-AAC TTT GTG CAG GCC ATG ATG-3′ R: 5′-GGC AGA AGA TGG CGG TCG-3′
*CPT2*	F: 5′-GCC TCT CTT GGA TGA CAG C-3′ R: 5′-CTG GTG TGC TTA TTC TGC T-3′

Abbreviations: *GAPDH*, Glyceraldehyde 3-phosphate dehydrogenase; *AMPK*, AMP-activated protein kinase; *SREBP1*, sterol regulatory element-binding protein1; *ACC*, acetyl-CoA carboxylase; *PPARα*, peroxisome proliferator-activated receptorα; *CPT1(2*), carnitine palmitoyltransperase1(2).

## Data Availability

The raw data are available from the corresponding author upon request.
